# Type I IFN signature in childhood-onset systemic lupus erythematosus: a conspiracy of DNA- and RNA-sensing receptors?

**DOI:** 10.1186/s13075-017-1501-z

**Published:** 2018-01-10

**Authors:** M. Javad Wahadat, Iris L. A. Bodewes, Naomi I. Maria, Cornelia G. van Helden-Meeuwsen, Annette van Dijk-Hummelman, Eline C. Steenwijk, Sylvia Kamphuis, Marjan A. Versnel

**Affiliations:** 1 Department of Immunology, Erasmus MC, University Medical Centre Rotterdam, 3015 CN Rotterdam, The Netherlands; 2 Department of Pediatric Rheumatology, Sophia Children’s Hospital, Erasmus MC, University Medical Centre, Rotterdam, The Netherlands

**Keywords:** Interferon type I, Childhood-onset SLE, TLR7, Cytosolic nucleic receptors, TBK1

## Abstract

**Background:**

Childhood-onset systemic lupus erythematosus (cSLE) is an incurable multi-systemic autoimmune disease. Interferon type I (IFN-I) plays a pivotal role in the pathogenesis of SLE. The objective of this study was to assess the prevalence of the IFN-I signature and the contribution of cytosolic nucleic acid receptors to IFN-I activation in a cohort of primarily white cSLE patients.

**Methods:**

The IFN-I score (positive or negative), as a measure of IFN-I activation, was assessed using real-time quantitative PCR (RT-PCR) expression values of IFN-I signature genes (*IFI44*, *IFI44L*, *IFIT1*, *Ly6e*, *MxA*, *IFITM1*) in CD14+ monocytes of cSLE patients and healthy controls (HCs). Innate immune receptor expression was determined by RT-PCR and flow cytometry. To clarify the contribution of RNA-binding RIG-like receptors (RLRs) and DNA-binding receptors (DBRs) to IFN-I activation, peripheral blood mononuclear cells (PBMCs) from patients were treated with BX795, a TANK-binding kinase 1 (TBK1) inhibitor blocking RLR and DBR pathways.

**Results:**

The IFN-I signature was positive in 57% of cSLE patients and 15% of the HCs. Upregulated gene expression of TLR7, RLRs (*IFIH1*, *DDX58*, *DDX60*, *DHX58*) and DBRs (*ZBP-1*, *IFI16*) was observed in CD14+ monocytes of the IFN-I-positive cSLE patients. Additionally, RIG-I and ZBP-1 protein expression was upregulated in these cells. Spontaneous IFN-I stimulated gene (ISG) expression in PBMCs from cSLE patients was inhibited by a TBK1-blocker.

**Conclusions:**

IFN-I activation, assessed as ISG expression, in cSLE is associated with increased expression of *TLR7*, and RNA and DNA binding receptors, and these receptors contribute to IFN-I activation via TBK1 signaling. TBK1-blockers may therefore be a promising treatment target for SLE.

**Electronic supplementary material:**

The online version of this article (doi:10.1186/s13075-017-1501-z) contains supplementary material, which is available to authorized users.

## Background

Childhood-onset systemic lupus erythematosus (cSLE) is a lifelong multi-systemic autoimmune disease that shares disease pathogenesis with adult-onset SLE but in most studies is characterized by a more severe disease course and poorer prognosis [1–3]. Interferon type I (IFN-I) plays a central role in the pathogenesis of SLE [4–7]. Surprisingly, trials blocking exogenous IFN-I or its receptor have shown limited effectivity so far, possibly due to our lack of knowledge of the pathways leading to IFN activation [8].

About half of patients with adult-onset SLE exhibit increased activation of IFN-I signaling or a so-called positive IFN-I signature [4, 5, 9]. This IFN-I signature is usually assessed by measuring IFN-I-stimulated gene expression. In a USA cohort of primarily non-white patients with cSLE with high disease activity, approximately 80–90% IFN-I activation has been reported [6, 10]. To our knowledge the prevalence of the IFN signature has not been studied in other cSLE cohorts.

**Fig. 1:**
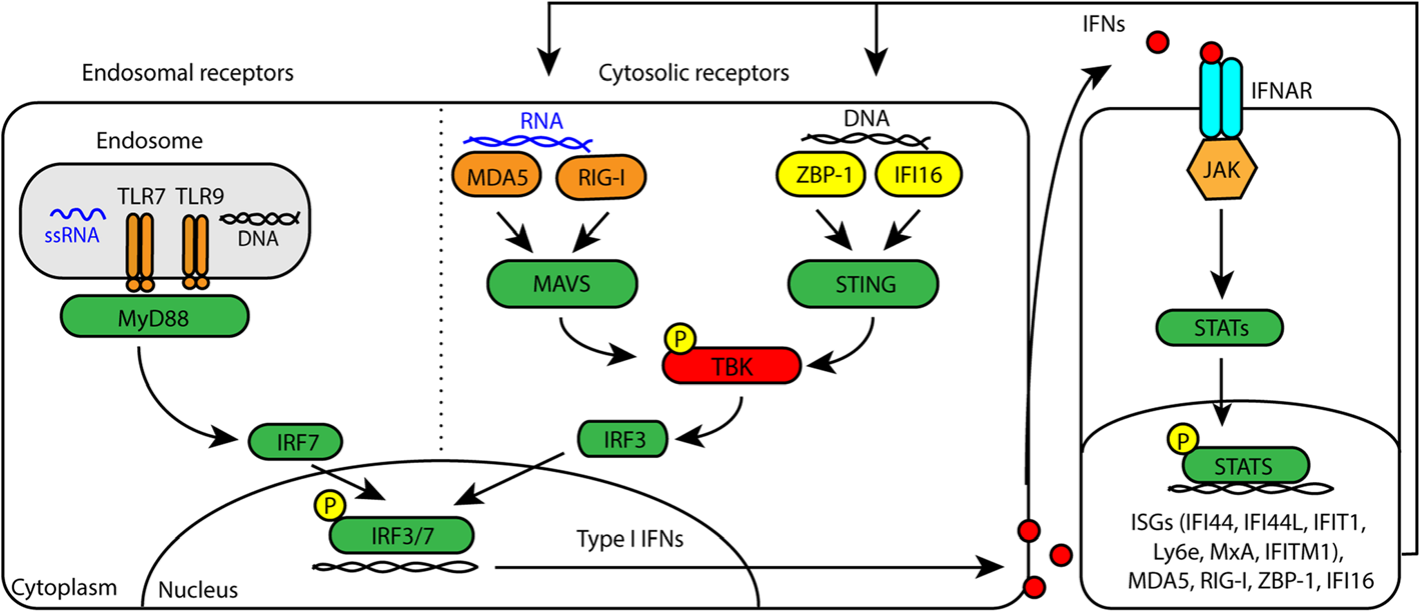
Simplified scheme of the induction of interferon (IFN) type I production by three signaling pathways: (1) endosomal receptors toll-like receptor (TLR)7 and TLR9; (2) RNA-binding cytosolic receptors MDA5 and RIG-I; and (3) DNA-binding receptors ZBP1 and IFI16. These pathways contribute to the activation of interferon regulatory factors (IRFs), which induce the expression of type I IFNs. Binding of IFN to cells that express the interferon alpha receptor (IFNAR) activates a cascade that leads to the expression of various IFN-stimulated genes (ISGs), known as the IFN type I signature

The endosomal toll-like receptors (TLRs) 7 and 9 induce IFN expression in response to internalized RNA-containing and DNA-containing immune complexes. Loss of the regulation of *TLR7* and *TLR9*, both binding exogenous self-nucleic acids, has been linked to SLE disease pathogenesis in mouse models and in humans [11, 12]. In addition to the TLRs, induction of IFN-I expression can also be initiated by two cytosolic nucleic-sensing receptor families, known as (i) the RIG-like receptors (RLRs) sensing RNA and (ii) the DNA-binding receptors (DBRs) (Fig. 1). In Sjögren’s syndrome we recently observed upregulation of RLRs that may contribute to IFN type I positivity in this disease [13]. The DBRs, like IFI16 and ZBP-1/DAI, bind intracellular double-stranded DNA (dsDNA) [14, 15] and as a result initiate production of IFN-I. Interestingly, mutations in the RLRs, DBRs and their downstream signaling molecules lead to systemic IFN-I activation in diseases grouped as “type I interferonopathies” [16–18]. Patients with these diseases have similarities to the SLE disease phenotype, pointing towards a central role of these molecules in IFN activation and potentially in the pathogenesis of SLE [16–18].

The objective of this study was to determine the prevalence of the IFN-I signature in a cohort of primarily white patients with cSLE and address the potential contribution of cytosolic nucleic acid receptors to IFN activation.

## Methods

### Patients and controls

Twenty-three patients with cSLE fulfilling at least four of the American College of Rheumatology criteria were recruited at the outpatient clinic of the department of pediatric rheumatology of the Sophia Children’s Hospital, Erasmus Medical Centre. Thirteen healthy controls (HCs), specifically checked for not having (viral) infections and not having family members with autoimmune diseases, were included. Patient characteristics are summarized in Table 1. The Medical Ethical Review Board of the Erasmus Medical Centre approved the study and written informed consent was obtained from all participants and their parents or legal guardians.

**Table 1:** Patient and control characteristics

		cSLE		
	HC (*n* = 13)	IFNpos (*n* = 13)	IFNneg (*n* = 10)	
Demographics				
Ethnicity				
White	13/13 (100%)	9/13 (69%)	7/10 (70%)	Ns^c^
Non-white	0/13 (0%)	4/13 (31%)	3/10 (30%)	Ns^c^
Gender				
Male (%)	3/13 (23%)	2/13 (15%)	2/10 (20%)	Ns^c^
Female (%)	10/13 (77%)	11/13 (85%)	8/10 (80%)	Ns^c^
Median age (years)	22 (15 ± 25)	15.8 (4.8 ± 18.2)	15.1 (9.3 ± 17.5)	Ns^a^
Disease duration (years)	-	0.85 (0 ± 3.4)	1.5 (0 ± 4.7)	Ns^b^
SELENA-SLEDAI	-	4 (0 ± 20)	3 (0 ± 13)	Ns^b^
Laboratory parameters				
ANA	-	12/13 (92%)	9/10 (90%)	Ns^c^
Anti-ds-DNA	-	4/13 (31%)	2/10 (20%)	Ns^c^
Anti-Ro52/Ro60	-	6/13 (46%)	0/10 (0%)	*p* = 0.019^c^
Anti-La	-	2/13 (15%)	0/10 (0%)	Ns^c^
Anti-RNP	-	5/13 (31%)	0/10 (0%)	*p* = 0.046^c^
C3 (g/l)	-	0.89 (0.3 ± 1.27)	1.1 (0.77 ± 1.72)	*p* =0.014^b^
C4 (g/l)	-	0.16 (0.02 ± 0.2)	0.19 (0.1 ± 0.37)	*p* = 0.049^b^
IgG (g/l)	-	10.3 (7.1 ± 27.6)	9.6 (8.4 ± 28)	Ns^b^
Medication (%)				
Hydroxychloroquine	-	10/13 (77%)	10/10 (100%)	Ns^c^
Mycofenolaatmofetil	-	3/13 (23%)	6/10 (60%)	Ns^c^
Prednisone	-	6/13 (46%)	5/10 (50%)	Ns^c^
Other medication	-	5/13 (38%)	5/10 (50%)	Ns^c^

Data are presented as median (IQR) or as number (%) of patients according to data distribution. Non-white ethnicity = Hindu and Suriname.

*SELEN*A Safety of Estrogens in Lupus National Assessment, *SLEDAI* Systemic Lupus Erythematosus Disease Activity Index, *ANA* antinuclear antibody, *Anti-RNP* antibodies to ribonucleoprotein, *C* complement, *IgG* immunoglobulin, *cSLE* childhood-onset systemic lupus erythematosus, *IFNpos* interferon (IFN) signature positive, *IFNneg* IFN signature negative, *HC* healthy control, *Ns* not significant

^a^Groups compared using the Kruskal-Wallis test (three groups)

^b^Groups compared using the Mann-Whitney U test (two groups)

^c^Groups compared using Fisher’s exact test (categorical data)

### Blood collection and isolation of monocytes and plasmacytoid dendritic cells

Blood samples were collected in sodium-heparin tubes (Greiner Bio-One, Germany) followed by isolation of peripheral blood mononuclear cells (PBMCs) as described before [19]. PBMCs were thawed, centrifuged for 5 min (1500 rpm, 4 °C) and resuspended in 100 μl sort‐buffer (PBS pH 7.4, 2 mM EDTA 1 M, 0.5% BSA). For membrane staining, cells were incubated for 15 min in the dark with anti‐CD14 (APC/Cy7; Becton Dickinson Biosciences, San Diego, USA), anti-BDCA‐4 (PE; Miltenyi Biotec, Bergisch Gladbach, Germany), anti‐CD123 (PE-Cy7; eBioscience, San Diego, USA), anti‐CD3 (PerCP-Cy5; Becton Dickinson Biosciences), and anti‐CD19 (APC; Becton Dickinson Biosciences). Cells were sorted using a FACSAria III cell sorter (BD Bioscience) and analyzed using FlowJo Sofware (TreeStar Inc., Ashland, USA).

### RT-PCR

RNAeasy columns (Qiagen, Hilden, Germany) were used to isolate total RNA followed by reverse-transcription to cDNA using a High-Capacity Reverse Transcription Kit (Applied Biosystems, Foster City, USA). RT-PCR analysis was performed using a 7900HT Fast Real-Time PCR System using predesigned primer sets (Applied Biosystems). Data were normalized to the expression of the household gene *ABL* to calculate the relative expression. *ABL* was previously described as a reliable household gene for myeloid cells [20]. *ABL* was not differentially expressed upon stratification of samples according to the IFN-stimulated gene expression scores (unpublished results). Fold-change values were determined from normalized cycle threshold (CT) values using the 2^-ΔΔCT method (User Bulletin, Applied Biosystems).

### Monocyte IFN type I signature and MxA protein assessment

Principle component analysis showed a subset of 6 genes (*IFI44*, *IFI44L*, *IFIT1*, *Ly6e*, *MxA*, and *IFITM1*) to explain more than 95% of the total variance of the 11 IFN-I-inducible genes tested. As the expression of the six IFN-I-inducible genes was not normally distributed, log expression values were log-transformed and IFN scores were calculated as described previously [19]. The mean and standard deviation (SD) of each IFN-inducible gene in the HC group was used to standardize expression levels of each gene for each study subject. Patients with cSLE were stratified into patients positive for the IFN-I signature (IFNpos; IFN score ≥10) and patients negative for the signature (IFNneg; IFN score <10). Flow cytometric analysis of MxA on CD14+ monocytes and the MxA-EIA was performed as previously described [21].

### Flow cytometric analysis of RLRs and DBR

Membranes were stained as described above with additional AnnexinV-BV421 staining (Milteny Biotec). Subsequently, cells were fixed and permeabilized by a permeabilization buffer set (eBioscience) with 1% paraformaldehyde, 0.5% saponin and stained with either rabbit anti-Mx1 (ProteinTech, Chicago, USA), rabbit anti-MDA5 (Abcam, Cambridge, UK), rabbit anti-DDX58 (Abcam), rabbit anti-IFI16 (Abcam) and rabbit anti-ZBP1 (Thermofischer, Rockford, USA)) and incubated in the dark for 45 min on ice. As a secondary antibody, chicken anti-rabbit-AF488 (Invitrogen, Carlsbad, USA) was used. Unstained cells and isotype-matched controls (Becton Dickinson Biosciences) were used to assess antibody specificity. Analysis was performed as previously described [21].

### In vitro stimulation bioassays

PBMCs were seeded at a concentration of 2 × 10E6/250 μL, and starved during 1 hour at 37 °C in RPMI with 0.5% fetal calf serum, 0.05% P/S. Cells were subsequently stimulated for 5 hours with 0.5 μg/mL Imiquimod (R837, IQ; InvivoGen, San Diego, USA), in the presence or absence of specific inhibitors for TANK-binding kinase 1 (TBK1)/IKKε (BX795, 1 μM, InvivoGen), TLR7 (IRS 661, 2 μM, TIB-Molbiol, Berlin, Germany) and TLR7 + TLR9 (ALX-746-255, 5 μM, Enzo Life Sciences, Lausen, Switzerland).

### Statistical analysis

The non-parametric Mann-Whitney U (two groups) and Kruskal-Wallis (three groups) tests were used to analyze comparisons between medians. The paired *t* test was used to compare means of paired data. Fisher’s exact test was used to compare categorical data. Spearman’s rho (rs) coefficient was calculated to assess correlation. Values of *p* < 0.05 were considered statistically significant. Graphpad Prism 5.0 (Graphpad Software, La Jolla, CA, USA) was used to design the graphs and IMB SPSS 20.0 (SPSS, Chicago, IL, USA) was used for the statistical analysis.

## Results

### Prevalence of the IFN-I signature in cSLE

**Fig. 2:**
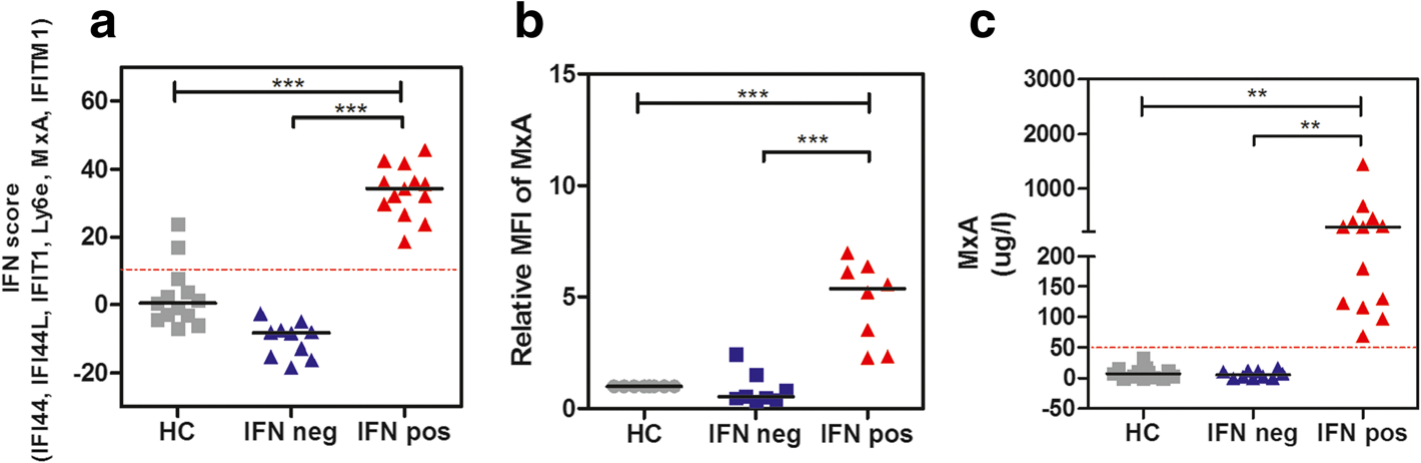
**a** Prevalence of the interferon (IFN) type I signature in patients with childhood-onset systemic lupus erythematosus (cSLE). Dotted line indicates the cutoff value of 10 for discrimination between IFN-negative (IFNneg) and IFN-positive (IFNpos) subjects. **b** Relative MxA expression was calculated as (MxA-specific staining patient (MFI)-isotype control patient (MFI))/(MxA-specific staining healthy control (HC) (MFI)-isotype control HC (MFI)). MxA is shown for HCs, IFN neg and IFN pos patients with cSLE. **c** MxA levels (ug/l) determined by MxA-enzyme immunoassays (EIA) in whole-blood lysates of HCs and patients with cSLE. Dotted line indicates the cutoff value of 50 for discrimination between IFN neg and IFN pos subjects. Every symbol represents one subject; horizontal lines describe the medians; groups were compared using one-way analysis of variance (three groups): *p < 0.05; **p < 0.01; ***p < 0.001; ****p < 0.0001

The IFN-I score was calculated for each subject by summing the standardized expression levels of the six IFN-I inducible genes. As there was a bimodal distribution of IFN-inducible genes in patients with cSLE, we set the threshold at an IFN-I score of 10. Using this threshold, 57% (13/23) of the patients with cSLE and 15% of the HCs (2/13) were IFN-I-positive (Fig. 2a). Previously we found that MxA protein expression assessed using flow cytometry on CD14^+^ monocytes and a whole blood enzyme immunoassay are applicable biomarkers for systemic IFN-I activation in Sjögren’s syndrome [21]. Both assays were tested simultaneously on the same PBMC samples. Results from these assays confirmed the results obtained by IFN-induced gene expression analysis (Fig. 2b, c).

### Increased expression of TLR7, RLR and DBR in CD14+ monocytes of cSLE

**Fig. 3:**
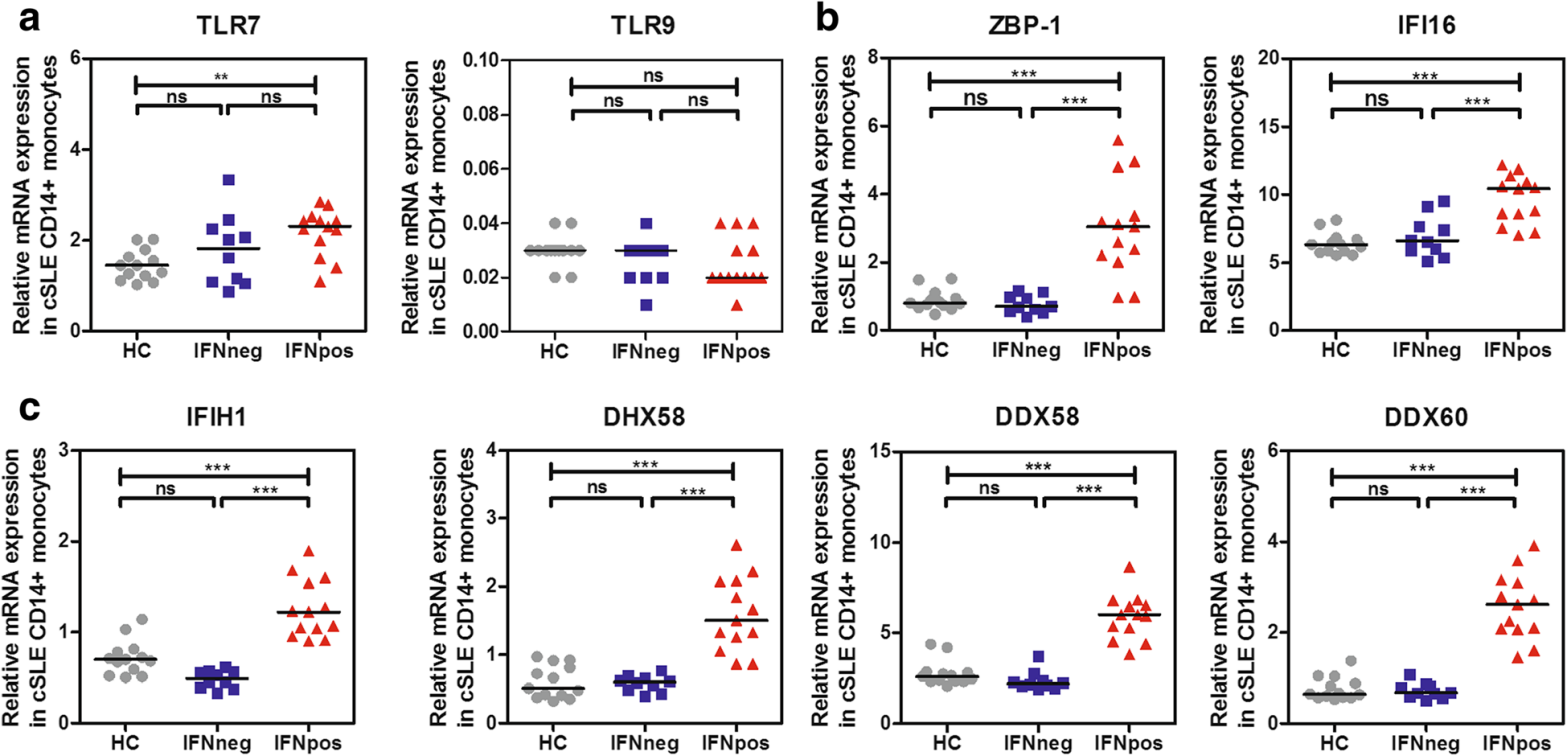
Upregulation of toll-like receptor 7 (*TLR7*) and cytosolic RNA-binding and DNA-binding receptors in interferon (IFN) type I positive (IFNpos) patients with childhood-onset systemic lupus erythematosus (cSLE). Relative mRNA gene expression of *TLR7* and *TLR9* (**a**), *ZBP-1* and *IFI16* (**b**) and *IFIH1*, *DHX58*, *DDX58*, and *DDX60* (**c**) in CD14+ monocytes from patients with cSLE (*n* = 23) and healthy controls (HCs) (*n* = 13). Each symbol represents an individual sample; horizontal lines represent medians. One-way analysis of variance was used to compare the three groups: Ns, not significant; *p < 0.05; **p < 0.01; ***p < 0.001; ****p < 0.0001. *IFNneg* IFN-negative patients, *IFNpos* IFN-positive patients

Upon ligand binding the TLRs, RLRs and DBRs all initiate IFN-I production (Fig. 1). The gene expression of *TLR7*, *TLR9*, four RLRs and two DBRs was assessed in CD14+ monocytes from patients with cSLE stratified into IFNpos and IFNneg patients. *TLR7* expression was significantly upregulated in IFNpos patients compared to HCs (Fig. 3a). There were no significant differences in *TLR7* expression between IFNneg and IFNpos patients or between IFNneg patients and HCs. In addition, *TLR9* expression did not differ between the groups.

The expression levels of the RLRs *IFIH1*, *DHX58*, *DDX58* and *DDX60* and the DBRs *ZBP-1* and *IFI16* were significantly upregulated in IFNpos patients compared to IFNneg patients and HCs (Fig. 3b, c). There was no significant difference between IFNneg patients and the HCs in RLR or DBR expression levels. Furthermore, expression levels of the RLRs and DBRs were positively correlated with IFN scores (Additional file 1).

### Increased RIG-I and ZBP-1 protein levels in cSLE

**Fig. 4:**
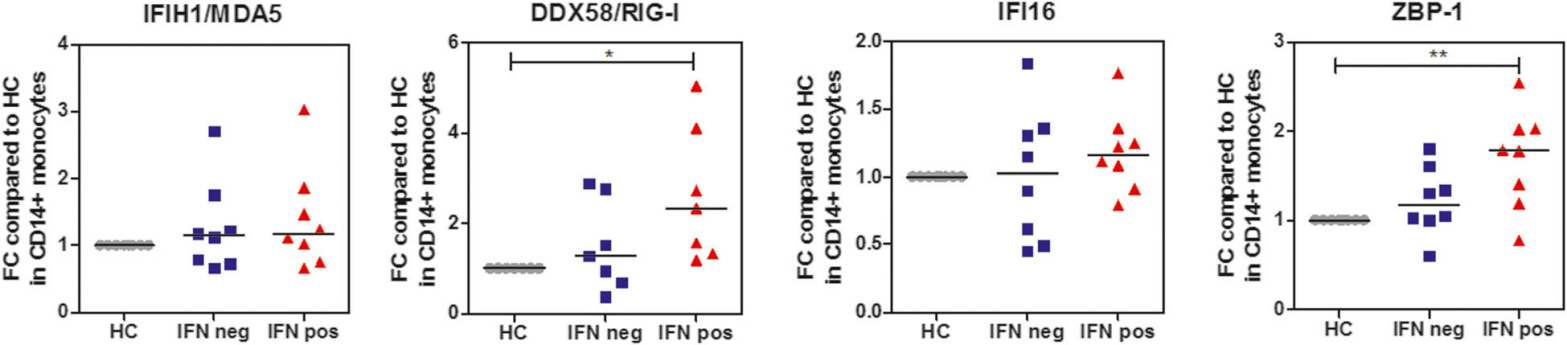
Upregulated protein expression of RIG-I and ZBP-1 in interferon (IFN)-positive CD14+ monocytes from patients with childhood-onset systemic lupus erythematosus (cSLE). Flow cytometric analysis of MDA5, RIG-I, IFI16 and ZBP-1 in CD14+ monocytes from IFN-positive (IFNpos) patients with cSLE (*n* = 8), IFN-negative (IFNneg) patients with cSLE (*n* = 8) and healthy controls (HCs) (*n* = 8). Each symbol represents an individual sample. The Kruskal-Wallis test was used to compare the three groups. Data are represented as fold change (FC) compared to HCs: *p < 0.05; **p < 0.01; ***p < 0.001; ****p < 0.0001

To study protein expression of RLRs and DBRs we performed flow cytometric analysis of MDA5, RIG-I, IFI-16 and ZBP-1 expression in CD14+ monocytes from IFNpos and IFNneg patients with cSLE and from HCs. The gating strategy and a representative figure are depicted in Additional file 2. RIG-I and ZBP-1 protein expression was significantly upregulated in CD14+ monocytes from IFNpos patients with cSLE compared to HCs (Fig. 4). There were no significant differences in MDA5 and IFI16 protein levels in CD14+ monocytes from patients and HCs. Plasmacytoid dendritic cells (pDCs) are known to upregulate RLRs and DBRs upon IFN-I activation. In pDCs from IFNpos patients with cSLE the expression of ZBP-1 and IFI16 was significantly upregulated (Additional file 3).

### TBK1/IKKε inhibitor blocks IFN-I activation in PBMCs from patients with cSLE

**Fig. 5:**
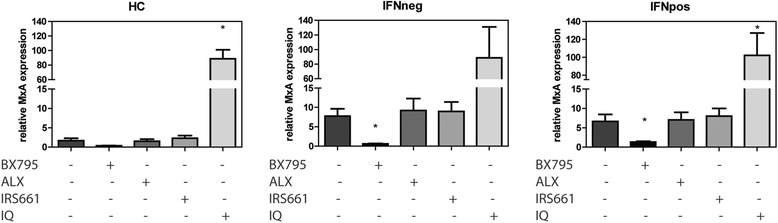
TANK-binding kinase 1 (TBK1)/inhibitor of nuclear factor kappa-B kinase subunit epsilon (IKKε) inhibits interferon (IFN) type I activation in peripheral blood mononuclear cells (PBMCs) from patients with childhood-onset lupus erythematosus (cSLE). Relative MxA gene expression after 5-h culture of PBMCs from healthy controls (HCs), IFN-negative (IFNneg) or IFN-positive (IFNpos) patients with cSLE stimulated with imiquimod (IQ) (1 μg/ml) or incubated with TBK1/IKKε inhibitor (BX795)(1 μM), toll-like receptor (TLR)7 and TLR9 inhibitor (ALX-746-255) (2 μM) or TLR7 inhibitor (IRS661) (5 μM). Unstimulated cells and cells without inhibitors added were cultured in starvation medium and used as control for baseline IFN activation. Gene expression data are presented as means ± SEM of four independent experiments (*n* = 5 per group). Means were compared to starvation medium using the paired *t* test: *p < 0.05; **p < 0.01; ***p < 0.001; ****p < 0.0001

To study the contribution of the RLR and DBR pathways to IFN-I activation in cSLE we blocked these pathways using a TBK1/IKKε inhibitor (BX795). A titration of BX795 is shown in Additional file 4. TLRs were blocked with inhibitors for *TLR7* (IRS661) [13] and TLR7 + TLR9 (ALX-746-255). As a positive control for the effectivity of the blockers, HC PBMCs were stimulated with the TLR7-agonist imiquimod (IQ) to induce IFN-I positivity followed by incubation with these inhibitors (Additional file 5). PBMCs from IFNpos and IFNneg patients with cSLE, without any additional stimulation, exhibited increased spontaneous IFN-stimulated gene expression compared to HCs as determined by MxA expression (Fig. 5). Incubation with the TBK1/IKKε inhibitor completely reduced the spontaneous IFN-I stimulated gene expression in cells from patients with cSLE. Inhibition of TLR7 or TLR7 + TLR9 had no effect on the intrinsic spontaneous IFN activation in PBMCs of IFNneg and IFNpos patients with cSLE (Fig. 5).

## Discussion

This study shows increased expression of TLR7 and the cytosolic receptors of the RLR and DBR families in monocytes of IFN-I-positive patients with cSLE. Blocking of the RLR and DBR signaling pathway downregulated IFN-I-stimulated gene expression indicating a contribution of these receptors to systemic IFN-I activation in SLE.

In our cohort of patients with cSLE, 57% had a positive IFN-I signature. This is in line with our earlier observations in a cohort of patients with adult-onset SLE [9]. Previous studies report that 80–90% of patients with cSLE are IFN-I-positive; these were primarily non-white patients with relatively high disease activity [6, 10]. In contrast, our cohort consists of mainly white patients with cSLE of low disease activity, which may account for at least part of the difference in prevalence, as the presence of IFN-I signature is related to disease activity [19]. As in the other cSLE cohorts, most patients with cSLE in our cohort used anti-inflammatory medication. The presence of an IFN-I signature in patients receiving medication indicates that current treatments are not able or are only partly able to downregulate IFN-I stimulated gene expression.

Upon stratification of IFNpos and IFNneg patients with cSLE, we identified upregulation of TLR7 in IFN-positive cSLE. This supports a role for TLR7 in the induction of IFN-I activation in SLE as has been demonstrated in animal models [22, 23]. Interestingly, a Mexican cohort of patients with cSLE showed that the gene dosage of TLR7 is an important risk factor for cSLE susceptibility [24]. In our ex vivo experiments, TLR7 or TLR7 + TLR9 inhibitors did not decrease IFN-I activation in patients with cSLE. This is likely due to the short culture period of 5 hours, which does not allow formation of nucleic acids containing immune complexes, which are required for TLR7/9-driven IFN induction. Therefore, the exact role of TLRs in comparison with cytosolic receptors remains to be established.

The expression of cytosolic receptors belonging to the RLRs and DBRs, was upregulated in IFNpos patients with cSLE compared to HCs and IFNneg patients. Accumulating evidence indicates an important role for aberrancies in these receptors and their downstream signaling molecules in monogenic diseases with clinical similarities to SLE [16, 17]. Interestingly, a recent study showed correlation between IFN-I activation and the expression of an endogenous virus-like genomic repeat element L1 in kidney tissue of patients with lupus nephritis. As such an L1 element activates RLRs this supports a role of this receptor family in SLE [25].

The potential contribution of RLRs and DBRs to IFN-I activation was also supported by our ex vivo experiments showing clearly decreased IFN-I stimulated gene expression in all patients with cSLE upon ex vivo blocking of TBK1. TBK1 is at the crossroad downstream of the RLR and DBR signaling pathways. Interestingly, TBK1 upregulation has been observed in PBMCs from patients with SLE [26] and inhibition of TBK1 activity suppresses IFN-I induced autoimmunity in a mouse model of SLE [27]. Blockade of TBK1/IKKε with BX795 was also found to inhibit IFN-I-stimulated gene expression in PBMCs from a patient with a gain-of-function mutation in STING, which resulted in over-secretion of IFN-I [28]. In our ex vivo experiments, PBMCs from IFNneg patients also had higher spontaneous intrinsic IFN-I-stimulated gene expression, which could be decreased by TBK1 inhibition with BX795. This is probably due to stimulation of the IFN-I-inducing pathways by the presence of more dead cells and cell material, which we always observe in samples from patients with SLE compared to controls, despite the same isolation procedure. This is in line with data showing greater vulnerability of the cells in SLE [29].

To date, inhibiting IFN activation by blocking IFN-I receptor (IFNAR) by biological agents so far has had encouraging results but the treatment is only effective in a subset of the patients [30]. More upstream interference using TBK1 inhibitors to prevent the induction of IFN expression might be a better approach. With TBK1 as an upstream signaling hub inducing IFN-I expression and more than 20 patented TBK1 inhibitors already developed, a novel treatment target for clinical applications might enter the field. Compared to most other biological agents, small-molecule TBK1 inhibitors have two advantages: (1) the inhibitors can be taken orally and (2) they are expected to have fewer side effects due to the high specificity [27].

This study has limitations. All patients are receiving treatment, which could have affected the IFN-I activation and due to lack of reliable assays to detect systemic IFN-I activation in serum, the IFN-induced gene expression was used.

Several studies in PBMCs of adult SLE patients describe a difference between IFN-α or IFN-β induced genes [7, 31]. We did not make this distinction in our study as we used monocytes and these IFN-subtype-specific-induced genes can differ per cell type. Additionally, the treatment presently tested in clinical trials is focused on blocking the IFNAR, which binds both IFN-α and IFN-β.

Furthermore, we studied mRNA and protein expression from monocytes but the patient’s pDCs were only studied by flow cytometry. However, monocytes are considered important responders to RLR and DBR triggering and the ex vivo cultures of PBMCs from patients simulate the in vivo situation. The TBK1 inhibitor used also inhibits IKKε. Therefore a role of IKKε in the IFN-I activation in SLE cannot be excluded.

## Conclusions

Overall, the IFN-I signature was present in 57% of patients with cSLE and was associated with increased expression of TLR7 and cytosolic nucleic acid binding receptors. These RLRs and DBRs contributed to the spontaneous ex vivo IFN-I-stimulated gene expression via TBK1 signaling. Inhibitors of TBK1 are therefore a promising treatment target for SLE.

## Additional files

Additional file 1: Correlation between RLR or DBR expression levels and IFN scores. (PDF 345 kb)

Additional file 2: Gating strategy and representative histogram. (PDF 281 kb)

Additional file 3: RLR and DBR protein expression in pDCs from patients with cSLE. (PDF 204 kb)

Additional file 4: Titration curve for BX795. (PDF 178 kb)

Additional file 5: Effectivity of inhibitors of TBK1, TLR7 and TLR7 + TLR9 to downregulate imiquimod-induced MxA expression. (PDF 248 kb)

## Abbreviations

BSA: Bovine serum albumin
cSLE: Childhood-onset systemic lupus erythematosus
CT: Cycle threshold
DBR: DNA-binding receptor
HC: Healthy controls
IFNAR: IFN type I receptor
IFN-I: Interferon type I
IKKε: Inhibitor of nuclear factor kappa-B kinase subunit epsilon
IQ: Imiquimod
PBMCs: Peripheral blood mononuclear cells
PBS: Phosphate-buffered saline
pDCs: Plasmacytoid dendritic cells
RLR: RIG-like receptor
RT-PCR: Real-time quantitative PCR
SD: Standard deviation
TBK1: TANK-binding kinase 1
TLR: Toll-like receptor
