# Supplementary material for: Type I IFN signature in childhood-onset systemic lupus erythematosus: a conspiracy of DNA- and RNA-sensing receptors?
Source: Arthritis Res Ther. 2018 Jan 10;20:4. doi: 10.1186/s13075-017-1501-z (PMC5763828; doi:10.1186/s13075-017-1501-z)
Supplement: Supplementary file 1 — Correlation between RLR or DBR expression levels and IFN scores. (PDF 345 kb) [file 13075_2017_1501_MOESM1_ESM.pdf]

Additional file 1: Correlations of RLR and DBR expression levels with IFN scores

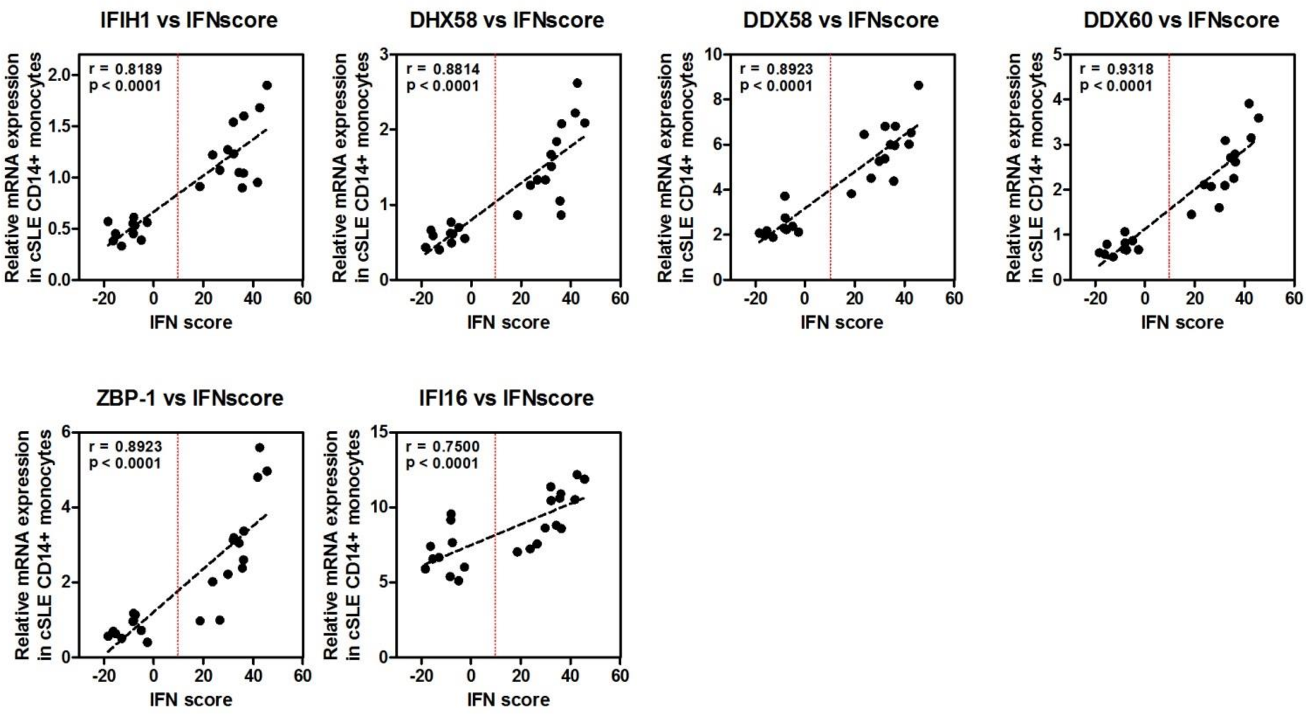

Additional figure S1: Correlation between interferon (IFN) type I score and mRNA expression of 6 IFN inducible genes (IFIH1, DDX58, DDX60, DHX58, ZBP-1, IFI16) in CD14+ monocytes in patients with cSLE (n=23). The correlation coefficients (r) and p values are shown. For correlations Spearman's ρ correlation test was used and to compare means the Mann–Whitney U test was used.
