# Supplementary material for: Type I IFN signature in childhood-onset systemic lupus erythematosus: a conspiracy of DNA- and RNA-sensing receptors?
Source: Arthritis Res Ther. 2018 Jan 10;20:4. doi: 10.1186/s13075-017-1501-z (PMC5763828; doi:10.1186/s13075-017-1501-z)
Supplement: Supplementary file 2 — Gating strategy and representative histogram. (PDF 281 kb) [file 13075_2017_1501_MOESM2_ESM.pdf]

Additional file 2: Gating strategy and representative histogram

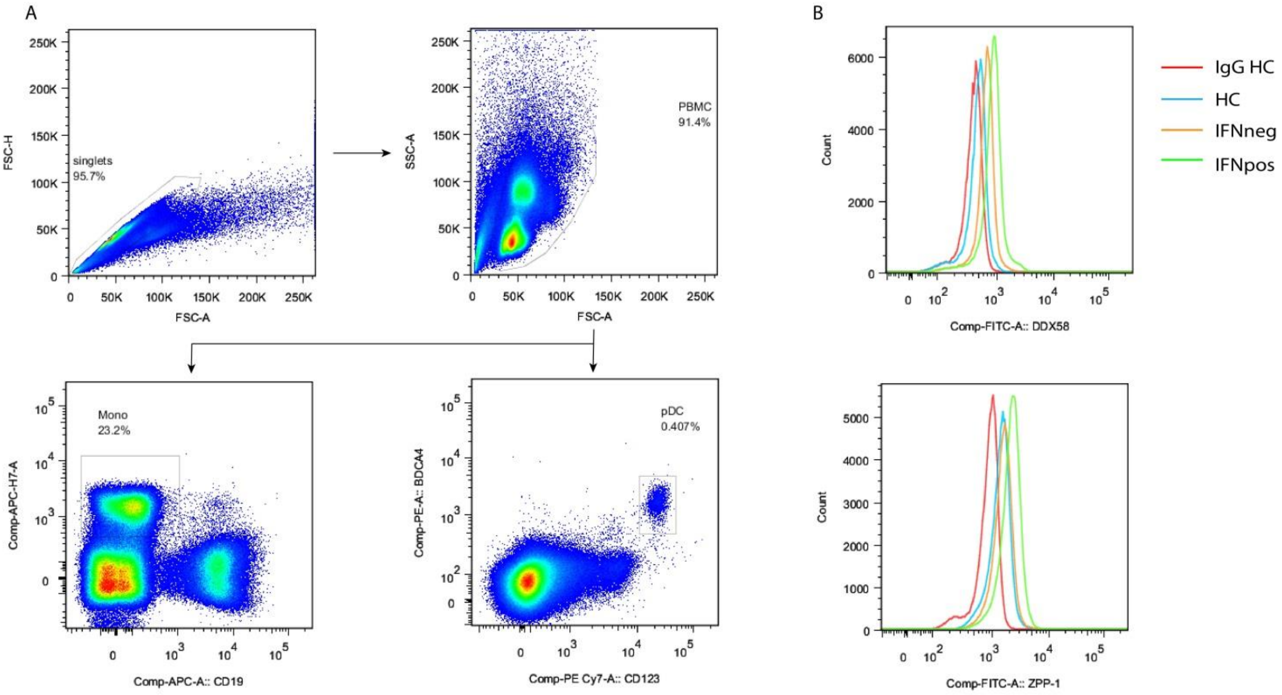

Additional figure S2: A) Gating strategy for CD14+ monocytes and CD123/BDCA4+ pDCs. PBMCS were gated after removal of doublets and dead cells. Within the PBMC fraction, CD14+ monocytes were gated and CD123/BDCA4+ pDCs **B)** representative histogram of protein expression levels (top: DDX58/RIG-I, bottom: ZBP-1).
