# Supplementary material for: Type I IFN signature in childhood-onset systemic lupus erythematosus: a conspiracy of DNA- and RNA-sensing receptors?
Source: Arthritis Res Ther. 2018 Jan 10;20:4. doi: 10.1186/s13075-017-1501-z (PMC5763828; doi:10.1186/s13075-017-1501-z)
Supplement: Supplementary file 3 — RLR and DBR protein expression in pDCs from patients with cSLE. (PDF 204 kb) [file 13075_2017_1501_MOESM3_ESM.pdf]

Additional file 3: RLR and DBR protein expression in pDC of cSLE patients

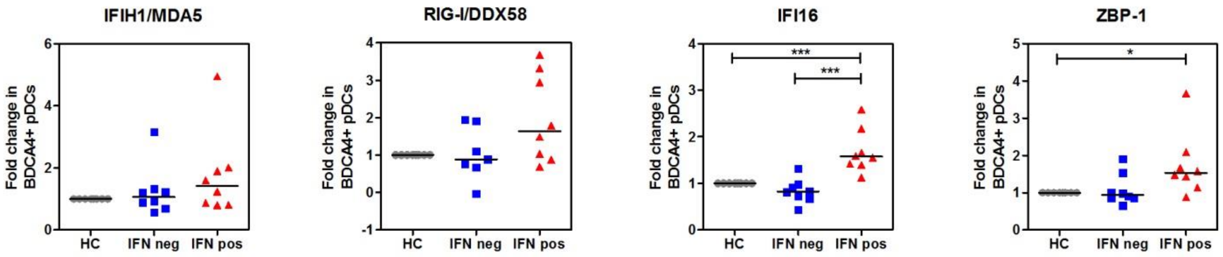

Additional figure S3: Flowcytometric analysis of MDA5, RIG-I, IFI16 and ZBP-1 in pDCs of IFNpos cSLE patients (n=8), IFNneg cSLE patients (n=8) and healthy controls (n=8). Each symbol represents an individual sample. To compare the three groups One-way ANOVA was used. Data represented in fold change compared to HC. *p<0.05; **p<0.01; ***p<0.001; ****p<0.0001.
