# Supplementary material for: Type I IFN signature in childhood-onset systemic lupus erythematosus: a conspiracy of DNA- and RNA-sensing receptors?
Source: Arthritis Res Ther. 2018 Jan 10;20:4. doi: 10.1186/s13075-017-1501-z (PMC5763828; doi:10.1186/s13075-017-1501-z)
Supplement: Supplementary file 4 — Titration curve for BX795. (PDF 178 kb) [file 13075_2017_1501_MOESM4_ESM.pdf]

Additional file 4: Titration curve of BX795

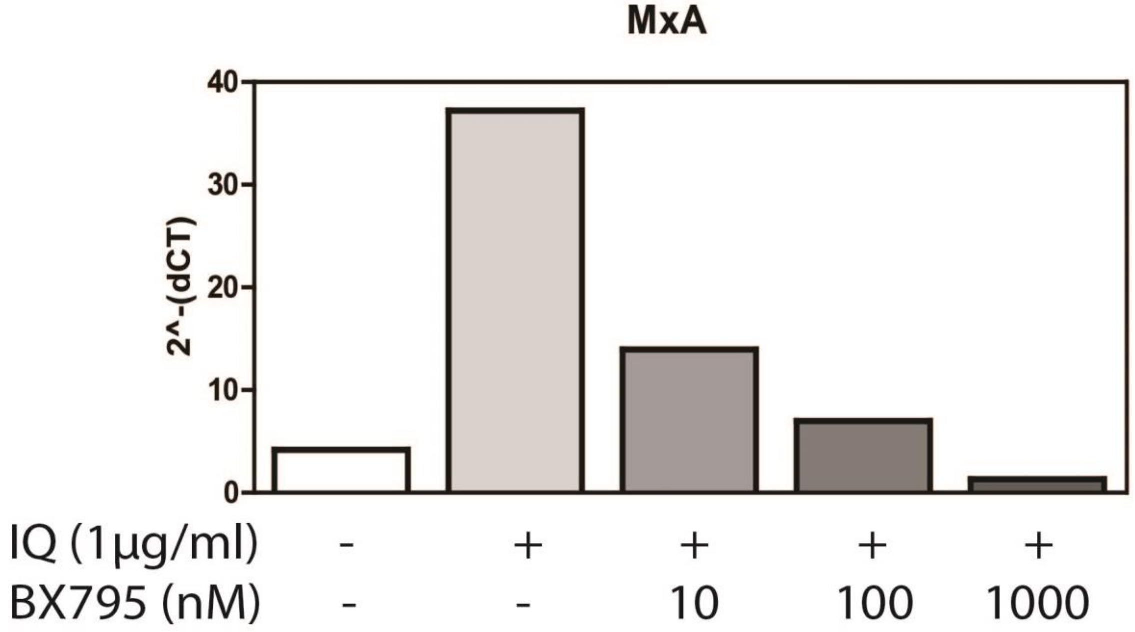

Additional figure S4: Titration of BX795 on HC PBMCs. Relative MxA gene expression after 5 hr culturing of PBMCs of HCs with imiquimod (IQ) (1 µg/ml)) and increasing amounts of TBK1/IKKε inhibitor (BX795)(10, 100 and 1000 nM). Gene expression data are presented as means ± SEM.
