# Supplementary material for: Type I IFN signature in childhood-onset systemic lupus erythematosus: a conspiracy of DNA- and RNA-sensing receptors?
Source: Arthritis Res Ther. 2018 Jan 10;20:4. doi: 10.1186/s13075-017-1501-z (PMC5763828; doi:10.1186/s13075-017-1501-z)
Supplement: Supplementary file 5 — Effectivity of inhibitors of TBK1, TLR7 and TLR7 + TLR9 to downregulate imiquimod-induced MxA expression. (PDF 248 kb) [file 13075_2017_1501_MOESM5_ESM.pdf]

Additional file 5: Effectivity of inhibitors for TBK1, TLR7 and TLR7+9 to downregulate Imiquimod induced MxA expression.

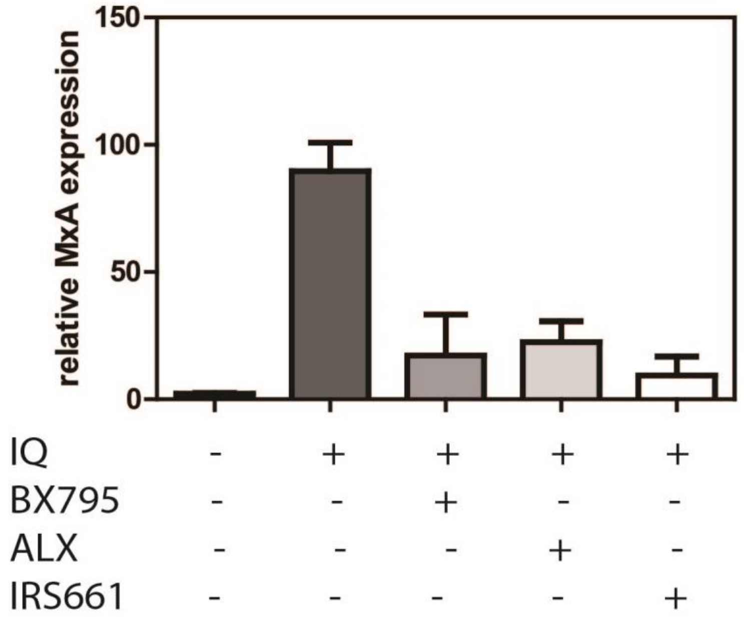

Additional figure S5 Relative MxA gene expression after 5 hr culturing of PBMCs of HCs with imiquimod (IQ) (1 µg/ml)) and/or incubated with TBK1/IKKε inhibitor (BX795)(1 µM), TLR 7 and 9 inhibitor (ALX-746-255) (2 µM) or TLR 7 inhibitor (IRS661))(5 µM). Cells without addition of stimuli or inhibitors are cultured in starvation medium and used as control for baseline IFN activation level. Gene expression data are presented as means ± SEM of 2 independent experiments.
